# Reflectance mapping with microsphere-assisted white light interference nanoscopy

**DOI:** 10.1038/s41598-024-77162-7

**Published:** 2024-11-06

**Authors:** Sébastien Marbach, Rémy Claveau, Paul Montgomery, Manuel Flury

**Affiliations:** ICube, Université de Strasbourg, CNRS, INSA, 67000 Strasbourg, France

**Keywords:** Microsphere, Nanoscopy, Resolution, White Light Interference Microscopy, Local Spectroscopy, Reflectance, Microscopy, Optical spectroscopy

## Abstract

The characterisation of novel materials presents a challenge that requires new and original developments. To face some of these demands for making measurements at the nanoscale, a new microsphere-assisted white light interference nanoscope performing local reflectance mapping is presented. This technique presents the advantages of being non-destructive, full-field and label-free. A 145 μm diameter microsphere, glued to the end of an optical fiber, is inserted inside the white light interference microscope to improve the lateral resolution from 940 nm to 520 nm. The acquisition and the Fourier transform processing of a stack of interference images superimposed on the virtual image produced by the microsphere allows the extraction of the local reflectance over a wavelength range of 460 nm to 900 nm and a field of view of 8 μm in diameter. The enhancement in the lateral resolution of the reflectance is demonstrated through the spectral distinction of neighboring ripples on a laser-textured colored stainless-steel sample that cannot be resolved without the microsphere, on regions with a surface of 279 × 279 nm^2^ horizontally spaced 279 nm apart. Future improvements could potentially lead to a lateral resolution of reflectance measurement over a 100 nm diameter area in air, paving the way to sub-diffraction reflectance mapping.

## Introduction

Optical spectrometers and ellipsometers are powerful characterization tools for bulk measurements, but with a lateral resolution^1^ limited to a few thousand µm^2^ at best. To study samples with microscopic dimensions, one solution developed is hyperspectral microscopy^2^. This technique is based on an optical microscope architecture combined with standard dispersive configuration^3^, a Fourier Transform spectrometer^4^, or even on white light interference microscopy^5–12^. However, hyperspectral microscopes present the same intrinsic limitations as optical microscopes, namely that of diffraction and are therefore unable to distinguish materials at the nanometer scale^13^.

Indeed, due to the diffraction of light, the lateral resolution of an optical microscope is theoretically limited to about half the illuminating wavelength, at best around 200 nm in the visible spectral domain. To overcome the diffraction barrier, new methods, classified as optical nanoscopes or super-resolved microscopes^14,15^, have been developed such as stimulated emission depletion microscopy (STED)^16^, photoactivated localization microscopy (PALM)^17,18^ or stochastic optical reconstruction microscopy (STORM)^19^. Nonetheless, these techniques require fluorophores, with the inherent risk of phototoxicity and photobleaching^13^. The contribution of scanning near-field optical microscopy (SNOM), from the study of surface nanostructures to spectral measurements should also be mentioned^15^. Other label-free super-resolved techniques have recently experienced rapid and significant development^14,20^, such as the promising technique of microsphere-assisted microscopy^21^.

Z. Wang et al. demonstrated in 2011 the super-resolution obtained with a standard microscope aided by a glass microsphere in air by observing 50-nm diameter nanopores spaced 50-nm apart using far-field imaging^21^. The microsphere creates a super-resolved magnified image, that is collected by the microscope objective, and recorded in the far-field by the camera. The gain in resolution has also been confirmed in immersive media^22^ with barium titanate (BTG) microspheres immersed in isopropyl alcohol^23^ or in PDMS^24^, and with 100-µm diameter BTG microspheres immersed in water^25^, the latter having been used to observe adenoviruses of about 75 nm in diameter. While there is debate concerning the exact explanation for the resolution improvement, it is now agreed that microspheres can achieve lateral resolutions of up to about λ/4-λ/5, i.e. about 100 nm in air with a white light source^26–28^. Moreover, microsphere-assisted microscopy is fairly easy to use and low cost, since the method only requires the positioning of the microsphere in the near field of the sample^21,29,30^.

Different proposals have been made to explain the origin of the super-resolution. For example, while modeling the microsphere with the photonic nanojet theory^26,31–33^ allows the determination of the position of the virtual image, it cannot explain a lateral resolution of better than λ/3. On the other hand, the conversion of evanescent waves into propagative waves by the microsphere is a promising hypothesis^27,34^, confirmed mathematically using the complex Snell-Descartes law^35^. But although the simulation shows the role of the whispering gallery mode, the physical origin of the conversion is not currently fully explained^31,36^.

The lateral resolution attainable and the field of view of the super-resolved image in microsphere-assisted microscopy, are directly linked to the diameter of the microsphere. The smaller the diameter, the better is the lateral resolution but the smaller the field of view^37,38^. Different solutions exist for manipulating the microsphere, for example using a micropipette^39^, an AFM cantilever^28,40^ or by a support attached to the objective^27,29,34^. They also allow an increase in the field of view thanks to scanning and stitching techniques, making the use of smaller microspheres possible to maintain a high resolution and avoid the tradeoff between the field of view and the gain in resolution, although at the cost of acquisition time. In addition, manipulation of the microsphere offers the possibility of performing contactless measurement^29^.

Microsphere-assisted microscopy also presents the advantage of being able to be used with several optical microscopy techniques such as dark-field microscopy^41^, fluorescence microscopy^24^, confocal microscopy^42^, and digital holographic microscopy^43^. Microspheres have also been successfully combined with interference microscopy to benefit both from the nanometric axial sensitivity and improved lateral resolution in nanometric topographic measurement, such as that of 3D sub-diffraction sized elements^40,44–48^. Recently, microspheres have also been used as a photonic nanojet generator to perform spectral measurements with a reduced lateral spot size of 210 nm^49^, by combining a microsphere with a reflection microscope and fibered spectrometer. This technique requires nonetheless lateral scanning of the sample to obtain spectral information over an area.

In the present work we propose combining the improved resolution imaging properties of the microsphere with white light interference microscopy to achieve local spectroscopic measurements with improved lateral resolution over a complete area.

This work is divided into four main sections: first, a short description of the setup used is proposed, before presenting the results. These are then further discussed in order to demonstrate the enhancement in lateral resolution of the reflectance information with microsphere-assisted white light interference nanoscopy. Finally, the material and methods are detailed.

## Results

### Experimental setup

**Fig. 1:**
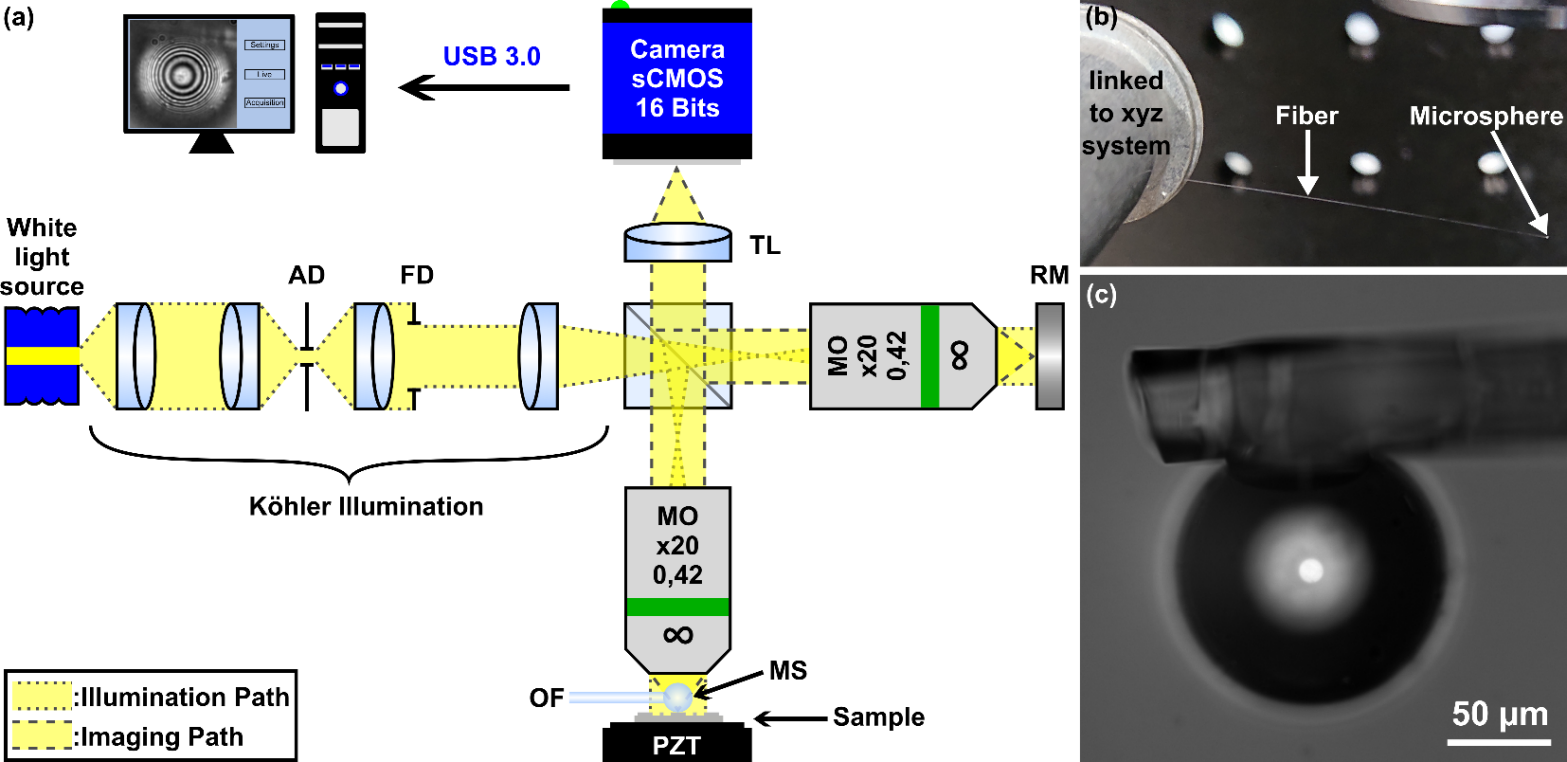
(**a**) Layout of the microsphere-assisted white light interference nanoscope. AD: Aperture Diaphragm. FD: Field Diaphragm. TL: Tube Lens. MO: Microscope Objective. RM: Reference Mirror. OF: Optical Fiber. MS: Microsphere. PZT: PieZo electric Translation table. (**b**) Image of the 145-µm diameter soda-lime glass microsphere glued to the end of an optical fiber. (**c**) Microscope image of the microsphere-optical fiber assembly.

A diagram of the setup used in this work is shown in Fig. 1(a). The system is a white light interference microscope in a Linnik configuration. To produce the phase shift between the object arm and the reference arm, the sample is placed on a piezo electric translation table (PZT). A soda-lime microsphere (MS), with a diameter of about 145 μm is optically glued to the end of the 50 μm-diameter core part of an optical fiber (OF), as illustrated in Fig. 1(b) and (c). The fiber-microsphere assembly is fixed to a mechanical xyz displacement system so as to be able to manipulate the microsphere and place it in contact with the sample at the required place. The same microsphere is used for the calibration of the interferometer and the spectroscopic measurement, to minimize the effects of the aberrations and artefacts produced by the microsphere.

More details about the materials and the acquisition method are given in the section at the end of the paper.

### Spatial resolution without and with the microsphere

**Fig. 2:**
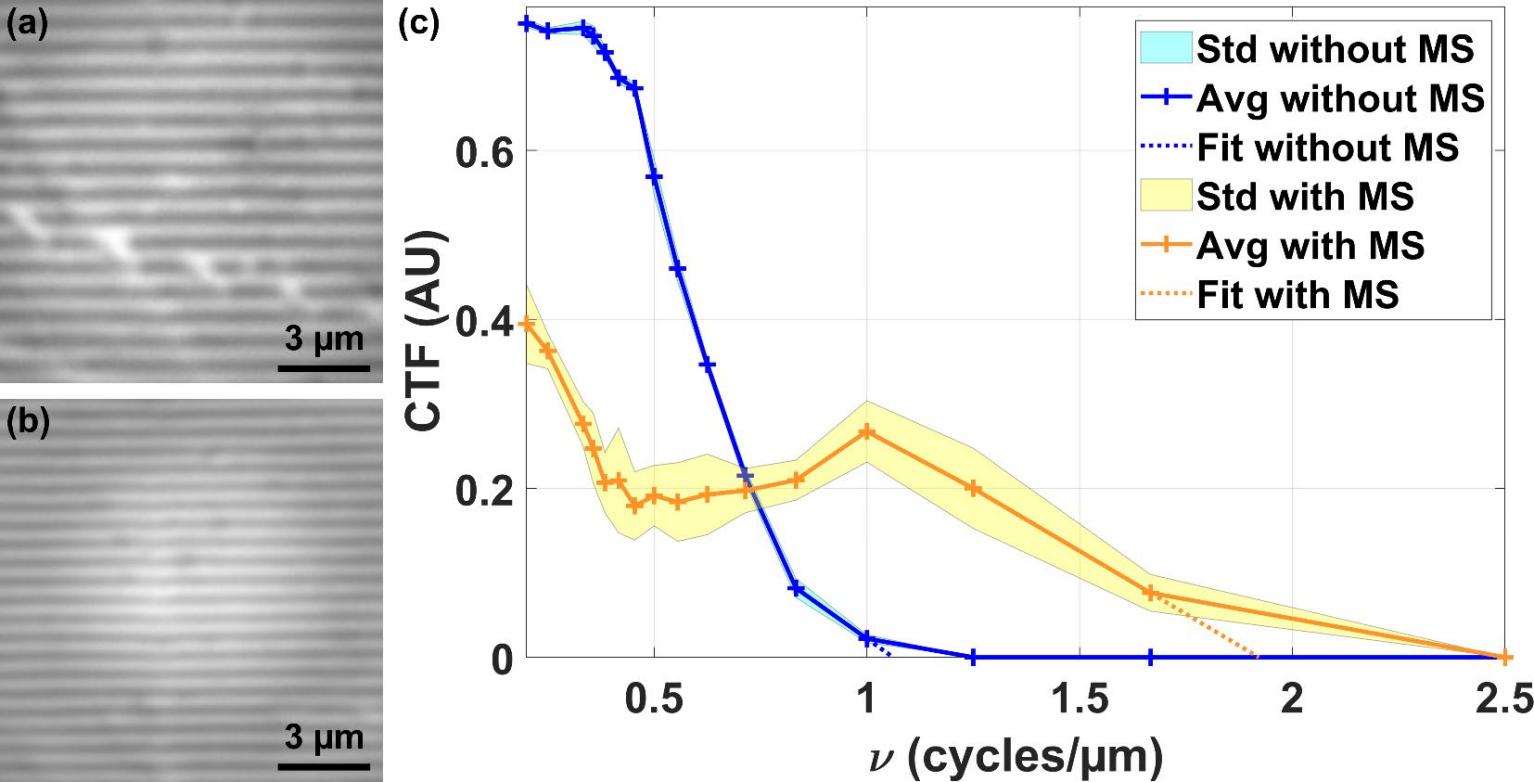
(**a**) 800 nm and (**b**) 600 nm period grating observed through the central part of the optical fiber-microsphere assembly. (**c**) Contrast Transfer Function (CTF) of the white light interference nanoscope used as a microscope (reference arm blocked) without (blue) and with (orange) assistance of the 145 μm microsphere glued on the optical fiber (MS). Std: Standard deviation. Avg: Average.

The Linnik interferometer used as a microscope, that is with the reference arm blocked off, is unable to image an 800 nm period grating without a microsphere. However, the introduction of the 145 μm-diameter microsphere allows the resolution of this 800 nm period grating and even the 600 nm period grating, as illustrated in Fig. 2(a) and (b), demonstrating an improvement in the lateral resolution. To quantify the gain in lateral resolution, the contrast transfer function (CTF) of the system is determined without and with the microsphere assistance as shown in Fig. 2(c). A “dome-like” shape is observable on the CTF measured with the microsphere. This shape is representative of the aberrations (principally spherical) introduced by the microsphere. Cut-off spatial frequencies of 1.06 cycles/µm and 1.92 cycles/µm, or lateral resolutions of 940 nm and 520 nm, are respectively obtained for the system alone and assisted by microsphere. The lateral resolution of the setup is thus improved by a factor of $\approx$1.8 when assisted with the 145-µm-diameter soda-lime glass microsphere.

The axial sensitivity of the Linnik interferometer evolves from about 1.5 nm without microsphere assistance to 3.5 nm with. The slight degradation in axial sensitivity is mainly due to the numerical removal of the topography of the microsphere, recorded simultaneously with the topography of the object (flat silicon substrate), and to the residual microsphere artefacts, non-perfect sphericity and imperfections. The axial sensitivity of the Linnik interferometer assisted with a 145-µm-diameter soda-lime glass microsphere enables depth measurements in the range of several nanometers.

### Proof of principle – reflectance measurements through the microsphere

**Fig. 3:**
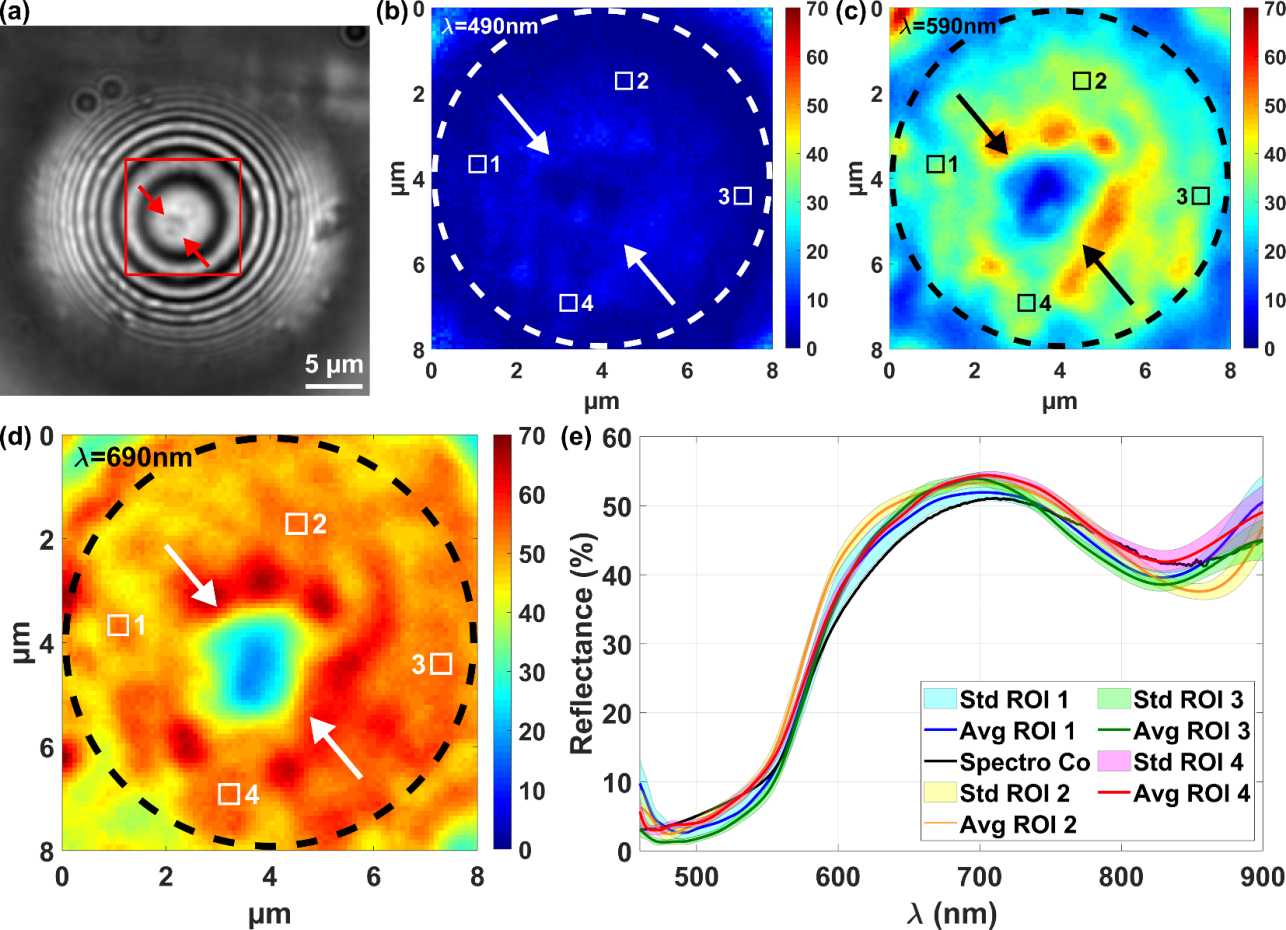
(**a**) Image through the microsphere in the interferometric mode. Reflectance maps extracted at the wavelengths of (**b**) 490 nm (**c**) 590 nm and (**d**) 690 nm from the spectral cube determined on the virtual image of the copper layer (Co) through the microsphere. (**e**) Reflectance extracted on the 4 regions (ROI) of 464 × 464 nm^2^. Std: Standard deviation. Avg: Average. The black line is the reflectance determined with a classical spectrometer.

To provide a proof of principle of spectral measurements through the microsphere, measurements were first made on a layer of copper deposited on a glass substrate using the previously described system, as shown with the interference image in Fig. 3(a). The signal processing described at the end of the paper results in a spectral cube, from which 3 reflectance maps at wavelengths of 490 nm, 590 nm and 690 nm have been extracted in Fig. 3(b), (c) and (d), covering an area of 8 × 8 µm^2^. As expected, the reflectance is higher at 690 nm, in the red part of the visible spectrum, than at 490 nm, in the blue-green part. The reflectance map at 490 nm (Fig. 3(b)) is fairly uniform, to within a few percent. At 590 nm and 690 nm (Fig. 3(c) and (d)), the region between the two arrows presents lower reflectance values. The most likely explanation for this is that a defect is present on the sample or inside the microsphere as visible between the two arrows on the interference image in Fig. 3(a). Since this defect was not present again on other measurements on different samples, it can be concluded that the defect does not come from an imperfection of the microsphere but is a feature of the sample. However, except for this defect, the reflectance spatial distribution is quite homogenous over a 8-µm diameter disk (represented by dashed circles in Fig. 3(b-d)). The surface area available for reflectance measurement is limited to the central region of the microsphere, due to the strong optical aberrations towards the edges. Choosing the four areas 1–4 of similar reflectance in Fig. 3(b-d), each of 5 × 5 pixels in size (464 × 464 nm^2^), the reflectance values in these zones are plotted as a function of the wavelength in Fig. 3(e). For comparison, the black line shows the measurement made on the same sample with a classical spectrometer, showing a good match with the measurements through the microsphere in the zones indicated over the wavelength range between 460 nm and 900 nm.

**Fig. 4:**
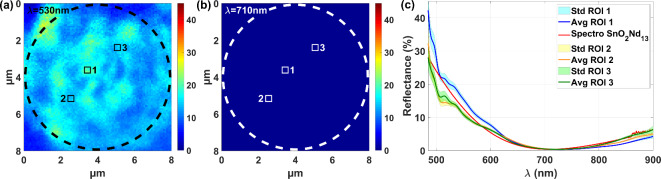
Reflectance maps at the wavelengths of (**a**) 530 nm and (**b**) 710 nm of the neodymium-doped tin dioxide sample (SnO_2_Nd_13_) through the microsphere. (**c**) Reflectance extracted on the 3 regions (ROI) of 464 × 464 nm^2^. Std: Standard deviation. Avg: Average. The red line is the reflectance determined with a classical spectrometer.

A second reflectance measurements through the optical fiber - microsphere assembly were achieved on a neodymium-doped tin dioxide sample. Reflectance maps at wavelengths of 530 nm and 710 nm, covering an area of 8 × 8 µm^2^, have been extracted in Fig. 4(a) and (b). The reflectance spatial distribution is fairly homogenous to within a few percent, without significant imperfections, over a 8 μm diameter disk (represented by dashed circles). The reflectance values, extracted from the 3 chosen ROI 1–3 (5 × 5 pixels^2^ in size, i.e. 464 × 464 nm^2^) inside the 8-µm diameter disk, are plotted as a function of the wavelength in Fig. 4(c). Again, the reflectances measured through the microsphere correspond well with the comparative reflectance determined with a classical spectrometer (red line).

These results demonstrate the reliability of the reflectance measurement through certain parts of the microsphere and serve as a proof of principle.

### Reflectance of ripples on laser-induced colored stainless-steel sample

**Fig. 5:**
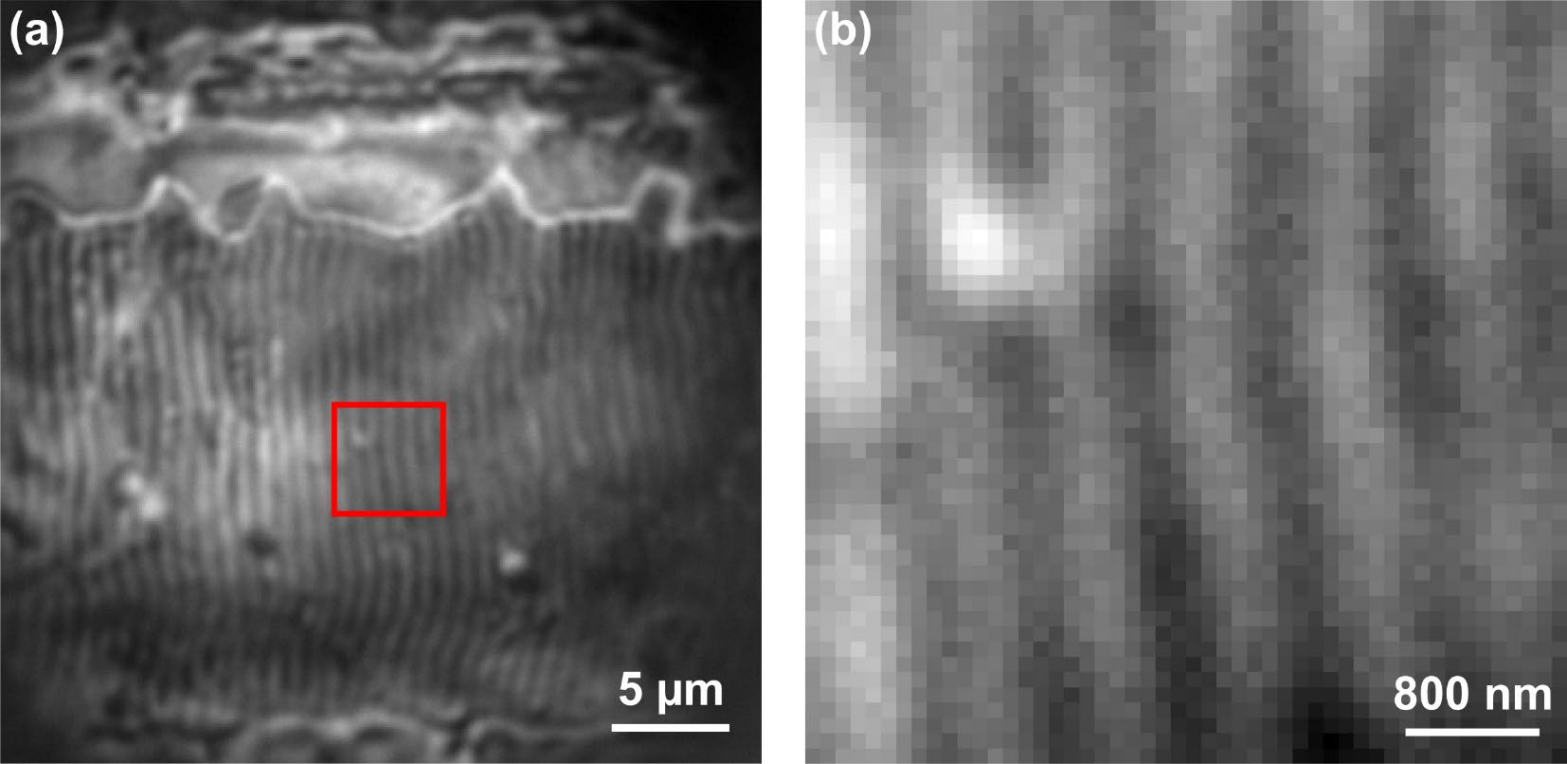
(**a**) Direct image of the surface roughness of a laser induced colored stainless-steel sample through the microsphere. Surface ripples are observable that are not resolved with the microscope objective alone. (**b**) Zoom of the red framed region in (a) in which interferometric measurements and reflectance analysis are performed.

Reflectance measurements were then performed through the microsphere placed on a turquoise part of a laser-induced colored stainless-steel sample. A direct intensity image through the microsphere with the reference arm blocked is shown in Fig. 5(a), revealing uniform and aligned ripples in the surface roughness that are not visible without the microsphere. This shows the enhancement in the lateral resolution through the microsphere.

**Fig. 6:**
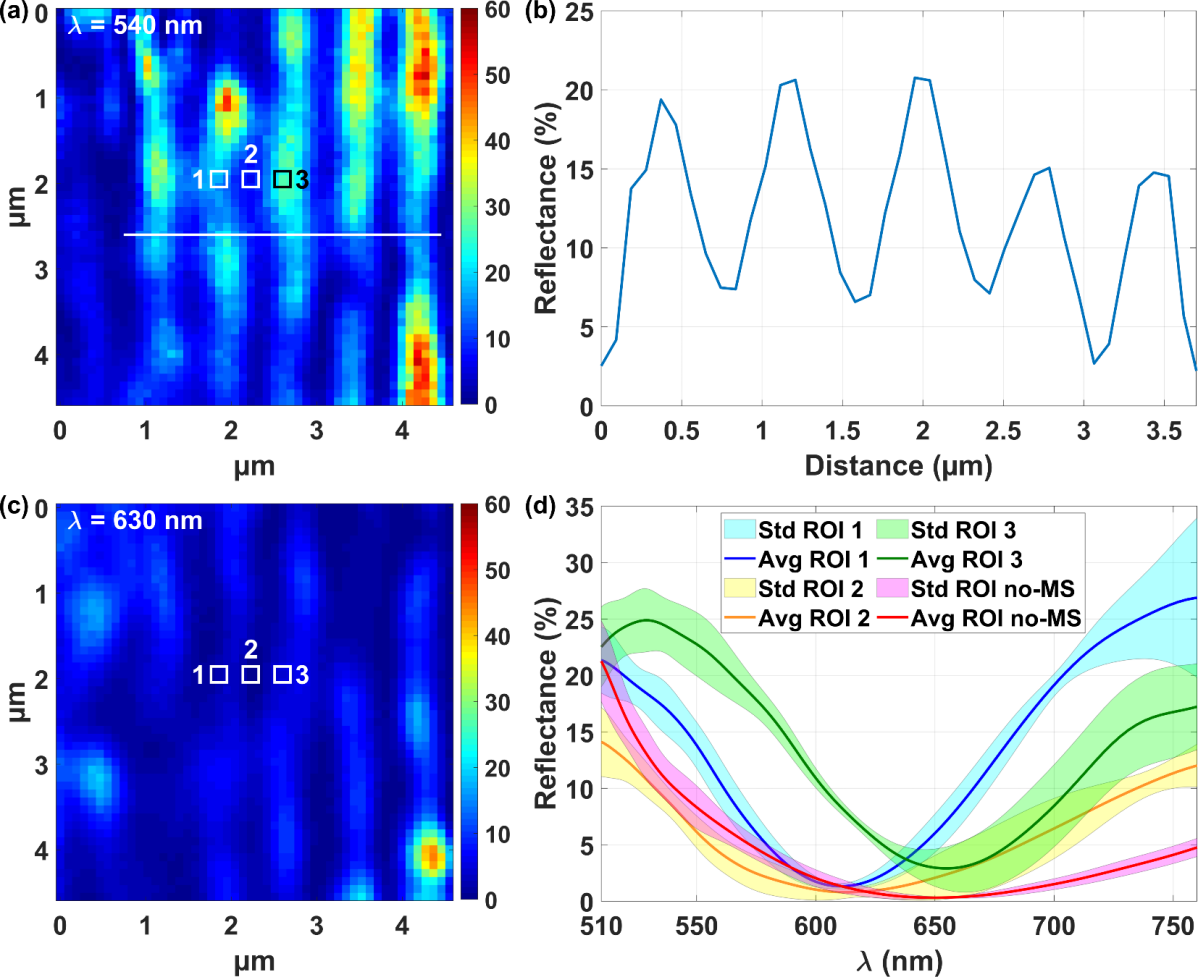
Reflectance maps at the wavelength of (**a**) 540 nm and (**c**) 630 nm of the laser induced colored stainless-steel observed through the microsphere. (**b**) Reflectance profile extracted along the white line plotted in (**a**). (**d**) Reflectance extracted on 3 regions (ROI) of 279 × 279 nm^2^, spaced horizontally of 279 nm apart, placed on successive undulations (not resolved without microsphere). The red line in the transparent magenta area is the reflectance measured without microsphere (no-MS) on 0.975 × 0.975 μm^2^ (3 × 3 pixels^2^). Std: Standard deviation. Avg: Average.

The reflectance study was then performed using the microsphere on the region framed in red in Fig. 5(a), in which a zoom is shown in Fig. 5(b). The results of the reflectance are shown in Fig. 6, with the ripples being recognizable on the reflectance map extracted at a wavelength of 540 nm in Fig. 6(a). The profile of the reflectance values along the white line from Fig. 6(a) is shown in (b), and shows that the reflectance varies across the ripples formed by the laser fabrication process. In the spectral map at 630 nm in Fig. 6(c), the reflectance of the ripples is lower than at 540 nm. This response is confirmed on the reflectance extracted in the three regions 1–3 shown in Fig. 6(c), that are 3 × 3 pixels^2^ in size (279 × 279 nm^2^) and separated horizontally by spaces of 279 nm, and plotted in (d). For comparison, the reflectance measured on 3 × 3 pixels^2^ without the microsphere^50^, i.e. on a larger surface area of 0.975 × 0.975 μm^2^ but without resolving the ripples, is also plotted in Fig. 6(d). The minor differences observed in reflectance are mainly due to the ability of the microsphere to distinguish the ripples, and thus to measure between them, whereas without the microsphere this spectral information is averaged, and therefore integrated together, since the ripples are not resolved. Other reasons may be a slight difference in location between measurements performed with and without the microsphere, and residual microsphere artefacts. However, the overall reflectance values measured through the microsphere are similar to those measured without a microsphere on the turquoise colored stainless-steel, demonstrating that the spectral information determined through the microsphere are representative of the sample. The variation in the reflectance values measured through the microsphere on the 3 different regions of the periodic patterns shows that the improvement in lateral resolution of the microsphere also apply to the reflectance measurements.

## Discussion

### Spatial resolution enhancement with the microsphere

According to the setup parameters (Numerical Aperture NA = 0.42, Effective Wavelength ${\lambda _{eff}}=720{\text{ nm}}$, AD open) and the Abbe criterion (${{{\lambda _{eff}}} \mathord{\left/ {\vphantom {{{\lambda _{eff}}} {(2NA}}} \right. \kern-0pt} {(2NA}})$), the best theoretical lateral resolution that could be reached would be equal to 857 nm. As expected, the experimentally determined lateral resolution of the microscope alone is slightly worse at 940 nm. However, when the 145 μm diameter microsphere is inserted into the microscope, the lateral resolution is improved to a value of 520 nm, much better than the theoretical limit: the diffraction limit is surpassed.

At the same time, the microsphere introduction only reduces the axial sensitivity from 1.5 nm to 3.5 nm: the Linnik interferometer assisted with the 145-µm-diameter microsphere thus enables the probing of depths with a sensitivity of several nanometers.

### Reflectance measurement through microsphere

The experimental measurements performed on the copper sample and the neodymium-doped tin dioxide sample demonstrate the possibility of measuring the reflectance over a wavelength range from 460 nm to 900 nm over a field of view having a diameter of about 8 μm, with microsphere-assisted white light interference nanoscopy. In this case, the size of the spectral analysis has been reduced using the microsphere to an area of 464 × 464 nm^2^ (5 × 5 pixels^2^). The reflectance cube determined through the microsphere of a turquoise-colored stainless-steel sample shows that the improvements in lateral resolution due to the microsphere is valid not only for imaging but also for reflectance measurements. The ripples are not resolved with the objective alone. With the microsphere, the ripples are resolved and their reflectance can be measured and distinguished on regions covering a surface of 279 × 279 nm^2^, spaced horizontally apart by 279 nm.

### Towards sub-diffraction reflectance mapping

This work extends the application of microspheres to a new domain in microscopy by making use of the lateral resolution enhancement to measure and map the reflectance of sub-diffraction sized structures. Indeed, the proposed technique paves the way to the development of methods responding to the needs in spectral characterization of nanostructures, such as metrology for semiconductor nanotechnologies or advanced studies of living matter. The use of a microsphere with a smaller diameter would improve the lateral resolution further^37,38^, in principle up to 100 nm in air^26,28^, and lead to a better localization of the reflectance information. Moreover, reflectance mapping with microsphere-assisted white light interference nanoscopy with our configuration presents the advantages of being non-destructive, full-field, label-free and with a modest cost.

However, if smaller microspheres are used to improve the lateral resolution, one disadvantage is that the field of view is smaller. To overcome the trade-off between the lateral resolution and the field of view, one strategy could be to combine the technique described in this paper with a lateral scan of the microsphere over the sample^27,40^, at the cost of longer acquisition and processing times. Another strategy to increase the field of view would be to spatially parallelise the acquisitions by replacing the microsphere with a matrix of microspheres^51^, although such a matrix is difficult to produce. These methods would enable to measure reflectance cubes with high spatial resolution on samples with large dimensions.

Another difficulty is the manipulation and the positioning of the microsphere in the near field of the object. In the present work this problem has been solved by gluing the microsphere to the end of an optical fiber and manipulating it with an xyz table into the required position in contact with the sample. To facilitate the spectral technique, a more robust and reliable method of manipulation needs to be developed, such as with a mechanical support system or even a microsystem. This would allow for instance placing the microsphere in the near field of the object without contact, although the thin-film effect between the bottom of the microsphere and the sample would have to be considered in the reflectance measurements. The thin film effect can also occur with samples with more complicated structures, for example with a height variation of the order of the effective wavelength, and would require a correction of the measured reflectance.

Finally, the introduction of a microsphere in the reference arm of the Linnik interferometer, similar to the microsphere in the object arm, could minimize microsphere aberration effects^48^, and therefore improve the quality and reliability of the reflectance measurement.

### Comparison with different label-free super-resolution technique

A brief comparison between the technique described in this paper and other selected label-free super-resolution techniques, at the time of writing and to the best of our knowledge, is proposed in Table 1.

**Table 1:** Comparison of label-free super-resolved techniques.

Technique	SNOM (Scanning Near field Optical Microscopy^52^)	Super-resolved Multispectral Lensless Ptychographic Microscopy^53^	TDM (Tomographic Diffractive Microscopy^54^)	Microsphere-Assisted White Light Interference Nanoscopy
Type of optics	Tip	Lensless	Microscopy	Microscopy
Lateral resolution	Up to 20 nm (depending on the tip used)	Half-pitch resolution of 550 nm (compared to 1.85 μm pixel size of the sensor)	Up to 75 nm (depending on illumination rotation and sample rotation)	Up to 100 nm in air (depending on microsphere nature and diameter)
Field of View	Point by point Requires scan to image up to several µm^2^	Wide field Up to some ≈ ten by ten cm^2^	Wide field Up to some ≈ ten by ten µm^2^	Wide field Up to some ≈ ten by ten µm^2^
Spectral information	Combinable with standard spectroscopy techniques	Spectral information on up to 6 wavelengths simultaneously	Complex refractive index at the measurement wavelength Requires a scan over wavelength	Able to measure quantitative reflectance from 460 nm to 900 nm
Acquisition and processing time	Typically several hours	About several minutes to hours	About several seconds to hours	About several minutes to hours
Other pros	Good axial sensitivity, up to about several nm	Aberration free Ease of use	Configurable for isotropic resolution (axial and lateral) of less than 200 nm	Good axial sensitivity, up to about several nm Quantitative reflectance
Other cons	Very long acquisition time Point by point method	Complex signal processing	Complex setup Restricted to low scattering sample	Sensitive to microsphere quality and aberrations

## Materials and methods

### Description of the white light interference nanoscope

A fibered halogen source (Ocean Optics HL-2000-HP, USA, $\Delta \lambda \approx 400{\text{ nm}}$) produces the white light of the interferometer, as shown in Fig. 1. The lab-made Köhler illuminator controls the properties of the incoming light. The Aperture Diaphragm (AD) can adjust the illumination cone reaching the sample, and the Field Diaphragm (FD) the field of view. The beam splitter divides the light into two beams, travelling through identical long working-distance objectives (MO, Mitutoyo M Plan Apo 20x/0.42, infinity corrected, Japan) in the object arm and the reference arm. These beams are respectively reflected by the sample and the reference mirror (RM). The second passage through the beam-splitter merge these beams, that are then imaged on the 16-bit cooled camera with a monochrome sCMOS sensor (PCO Edge 4.2 USB 3.0, Germany) thanks to the tube lens (TL). To make the fringes appear on the sample, the optical path difference between the arms is adjusted to be inferior to the coherence length (about 2–3 μm). A piezo electric translation stage (PZT, PI P621-ZCD, Germany) allows the movement of the sample, and thus to modify the optical path difference between the arms with nanometer precision. The camera and the PZT communicate through USB3.0 protocol with the computer and are controlled with a home-made LabVIEW (National Instruments, USA) program.

A soda-lime glass microsphere with a diameter of about 145 μm (MS, Cospheric SLGMS-2.5 125–150 μm, USA) has been attached to an optical-fiber (OF) consisting only of the core with a 50-µm-diameter. To do so, the end of the fiber was impregnated with optical glue (Thorlabs/Nordland NOA 68, USA) before placing it in contact with the edge of the microsphere deposited on a silicon substrate. The optical glue is then fixed with UV light to hold the microsphere on the optical fiber. The optical fiber holding the microsphere is mounted on a mechanical xyz translation table so as to be able to manipulate the microsphere in front of the objective (see the Fig. 1). The optical fiber - microsphere combination also allows to preserve the same microsphere for all the experiments, and consequently to perform a correct and accurate setup calibration and characterization. Some samples may contaminate or create impacts on the microsphere, leading to undesirable effects that may degrade the performance of the microsphere (reduced field of view, perturbed measurements, …). In this case, the best solution is to make a new optical fiber-microsphere assembly using a new and intact microsphere.

### System adjustment for use with microsphere

To adjust the system to perform correct measurement through the microsphere, the reference arm of the interferometer is first blocked off so as to be used as a simple microscope.

The microsphere in air and the region of interest of the sample are placed in the center of the field of view, and then brought into contact. By observing through the microsphere, the magnified virtual image of the sample with the best contrast is obtained. To determine the magnification due to the microsphere, a sample made of a contrast grating with a known period ${\kappa _{init}}$ is used. The period ${\kappa _{mic}}$ measured on the virtual image through the microsphere gives the magnification ${M_{mic}}$ thanks to the equation: ${M_{mic}}={{{\kappa _{mic}}} \mathord{\left/ {\vphantom {{{\kappa _{mic}}} {{\kappa _{init}}}}} \right. \kern-0pt} {{\kappa _{init}}}}$. With the microsphere glued onto the optical fiber, a magnification of about 3.75 is determined.

The reference arm is then unblocked, to use the system as a white light interference microscope. The insertion of the microsphere in the object arm introduces a difference in the optical path with that of the reference arm. The optical path difference must be compensated by translating the reference arm of the interferometer until the fringes appear on the virtual image of the sample through the microsphere. Correct interferometric measurements can then be performed through the microsphere.

### Contrast transfer function and spatial resolution determination

The contrast transfer function ($CTF$) is measured with the AD open and using a calibrated square contrast grating with known periods from 200 nm up to 5 μm in steps of 200 nm. The grating pattern consists of alternating lines of silicon (deposited) and glass (substrate) with a duty cycle of 0.5. For each grating with a spatial frequency $\upsilon$, the maximal and minimal intensities (${I_{max}}$ and ${I_{min}}$) are determined to obtain the contrast value thanks to Eq. (1).

1 $$CTF(\upsilon )=\frac{{{I_{max}}(\upsilon ) - {I_{min}}(\upsilon )}}{{{I_{max}}(\upsilon )+{I_{min}}(\upsilon )}}$$

To be more accurate, at least 10 profiles made of 5 patterns have been used to calculate the average (Avg) and the standard deviation (Std) value of the contrast in Fig. 2.

A linear numerical fit on the 2 last spatial frequencies resolved furnishes the dotted lines. The intersection between these lines and the null contrast value gives the cut-off spatial frequency and therefore the lateral resolution (equal to the inverse of the cut-off spatial frequency)^55^.

The axial sensitivity of the interferometer can be determined by evaluating the standard deviation of the topography of a flat sample^48^, for instance here a silicon substrate. To measure the topography of the sample, a stack of interference image is acquired (see below) and analysed with the Fourier Domain Analysis (FDA) algorithm^12,56,57^. When the sample is measured through the microsphere, the interference signal records the topography of the microsphere together with the topography of the sample. A numerical 2D polynomial fit is therefore performed on the result furnished by the FDA to remove the shape of the microsphere^44–48^ and finally obtained only the topography of the sample. As for reflectance measurement, only region about 8 × 8 μm^2^ centered in the middle of the microsphere are meaningful and analysed.

### Acquisition of one stack of interference images

Once the fringes appear on the image of the sample through the microsphere, the acquisition of a stack of interference images can be performed. To do so, the sample together with the microsphere is slightly defocused and then moved step by step with the PZT, so that the fringe pattern scans the whole of the depth of the sample. At each step, 10 images are captured and averaged to increase the signal to noise ratio of the resulting interference image recorded. During the measurement, i.e. the stack acquisition, the microsphere is no longer manipulated since the attractive force between the sample and the microsphere makes them move together and in harmony during the measurement. At each step of the PZT, the sample and the microsphere actually remain in contact, due to the force of attraction and the flexibility of the optical fiber, making them move simultaneously along the z-axis.

To obtain a good trade-off between the energy distribution, the data storage space and the acquisition time, the stack of interference images is acquired over a depth of 20 μm with a piezo displacement step of ${{{\lambda _{eff}}} \mathord{\left/ {\vphantom {{{\lambda _{eff}}} 8}} \right. \kern-0pt} 8}$, that is of 90 nm for the effective wavelength^50^ of the setup of 720 nm. This results in a stack of 228 interference images, with a size of up to 350 × 350 pixels^2^ (i.e. about 32 × 32 μm^2^ considering the microsphere magnification), that is acquired in less than 18 s with the LabVIEW program developed.

### Reflectance extraction theory, acquisition, and processing

The interferograms can be obtained by extracting the values over the depth Z at each pixel XY of the stack of interference images. The Fourier Transform ($\operatorname{FT}$) modulus of the interferogram then provides an “Effective Spectrum” (${S_{eff}}$), containing the reflectance of the sample multiplied by the spectral response of the microsphere and the interference microscope (depending on the reference mirror reflectance, and on the spectral response of the camera and optical components)^6,8^. The response of the whole instrument (interferometer and microsphere) is eliminated through a calibration step, where the interferogram (${I_{cal}}$) of a sample with a known reflectance (${R_{cal}}$) is measured with the same experimental conditions as those used to record the interferogram of interest (${I_{sam}}$). Assuming the object and the reference mirror reflectance to be constant with the angle of incidence of the illumination and negligible object scattering, the quantitative reflectance of the sample (${R_{sam}}$) is then inferred from Eq. (2), with $\delta$ corresponding to the optical path difference between the reference and the object arms ($\delta (z)=2z$ in reflection in air), and $k={{2\pi } \mathord{\left/ {\vphantom {{2\pi } \lambda }} \right. \kern-0pt} \lambda }$ corresponding to the wavenumber^8–12^.

2 $${R_{sam}}(k)={\left| {\frac{{\operatorname{FT} \left[ {{I_{sam}}(\delta )} \right]}}{{\operatorname{FT} \left[ {{I_{cal}}(\delta )} \right]}}} \right|^2} \cdot {R_{cal}}(k)={\left| {\frac{{{S_{eff,sam}}(k)}}{{{S_{eff,cal}}(k)}}} \right|^2} \cdot {R_{cal}}(k)$$

The reflectance obtained can be dilated as a function of the aperture diaphragm. To partially correct the dilatation, a beta-factor β has been defined^7^. This factor is equal to the ratio of ${\lambda _{max}}$ on ${\lambda _{eff}}$, with ${\lambda _{max}}$ corresponding to the wavelength at which the Fourier Transform of the interferogram is maximum. The corrected reflectance ${R_{corr}}$ is obtained with Eq. (3). (When the AD is closed, the beta-factor is equal to 1, when the AD is open, the beta-factor becomes bigger than 1).

3 $${R_{corr}}(\lambda )={R_{sam}}(\beta \lambda )$$

The averaging of the effective spectra extracted from several stacks of interference images improves the quality and the reliability of the reflectance information measured^12^. The spectral acquisition therefore consists in recording 10 successive stacks of interference images of the sample through the microsphere, leading to an increase in the acquisition time to about 3 min. The calibration step is also achieved by capturing 10 successive stacks of interference images on a silicon substrate through the microsphere. The optical fiber - microsphere assembly is the key element that allows the effects of the aberrations produced by the microsphere to be taken into account and reduced, and that prevents the introduction of other imperfections and artefacts that would occur if a different microsphere were used between the measurement step and the calibration step.

The numerical processing of the data is performed on MATLAB 2019b (MathWorks, USA) to compute every pixel in parallel. Due to optical aberrations towards the edge of the microsphere, only a region of about 100 × 100 pixels^2^, i.e. about 8 × 8 μm^2^ centered in the middle of the microsphere are meaningful and further analyzed. Every interferogram is apodised by a 15 μm-wide-Hamming window along Z to optimize the tradeoff between the spectral resolution and the sidelobe amplitude in the Fourier space. Indeed, according to Fourier Transform theory and Eq. (2), the reflectance is determined with a spectral resolution of $\Delta \lambda = 1.81 \cdot \lambda ^{2} /\left( {2L} \right)$ for a Hamming apodization window with a width L. For instance, Δλ = 15.1 nm at λ = 500 nm and Δλ = 38.6 nm at λ = 800 nm. A zero-padding on 1024 points followed by a linear interpolation are achieved to plot the reflectance with a 1 nm-step. The processing of the stack of interference images to provide a reflectance cube (x, y,λ), that is a stack of reflectance maps, takes less than 1 min with a computer equipped with an Intel i9 processor and 128 Go RAM.

### Sample information and setup settings

The copper sample was made by depositing a 2 μm thick layer of copper by evaporation on a glass substrate. The neodymium-doped tin dioxide sample (SnO_2_Nd_13_) was prepared by depositing neodymium-doped tin dioxide on a silicon substrate. Since these samples are used to demonstrate the reliability of the reflectance measurement through the microsphere, comparative reference reflectances were measured with a spectrometer Perkin Elmer Lambda 19 UV-VIS-NIR (USA). The interference measurements through the microsphere were performed with the aperture diaphragm closed, leading to β = 1 in Eq. (3).

The colored sample consisted of a 5 mm thick 316 L unpolished stainless-steel plate that had been processed with a LEM 40 system from Laser Cheval (France), a computer-controlled scanning laser beam system at a wavelength of 1064 nm. To obtain a turquoise laser-induced colored stainless-steel sample, the laser parameters were fixed to a pulse size of 1 µs and a frequency of 80 kHz while the sample was scanned line-by-line in air with a stage speed of 75 mm/sec. The reflectance measurement through the microsphere was conducted with the aperture diaphragm slightly open. A beta-factor of 1.07 was thus used in Eq. (3) to correct the reflectance dilatation. Consequently, the reflectance was accessible over a reduced wavelength range between 510 nm and 760 nm. The field of view was limited to about 4.5 × 4.5 μm^2^ due to some dust remaining on the edge of the view through the microsphere that prevented a correct calibration.

## Data Availability

The datasets generated during and/or analysed during the current study are available from the corresponding author on reasonable request.
